# Mid-Term Migration Behavior of an Uncemented Proximally Anchored Straight Stem—A Retrospective EBRA Migration Analysis

**DOI:** 10.3390/jcm12134335

**Published:** 2023-06-28

**Authors:** Philipp Blum, Johannes Neugebauer, Alexander Keiler, David Putzer, Julius Watrinet, Sebastian Biermeier, Dietmar Dammerer

**Affiliations:** 1 Department of Trauma Surgery, BG Trauma Center Murnau, 82418 Murnau, Germany; 2 Department of Orthopaedics and Traumatology, Krems University Hospital, 3500 Krems, Austria; 3 Karl Landsteiner Private University for Health Sciences, 3500 Krems, Austria; 4 Department of Orthopaedics and Traumatology, Medical University of Innsbruck, 6020 Innsbruck, Austria; 5 Department of Experimental Orthopaedics, Medical University of Innsbruck, 6020 Innsbruck, Austria

**Keywords:** total hip arthroplasty, Einzel Bild Röntgen Analyse, cementless, subsidence

## Abstract

Background: Aseptic loosening is one of the most-common causes of the failure of cementless stems. Einzel Bild Röntgen Analyse-Femoral Component Analysis (EBRA-FCA) allows the diagnosis of stem migration, which can be considered a factor in predicting implant survival. The current study aimed to present the migration behavior of a tapered proximally anchored straight stem. Methods: This retrospective study reviewed all consecutive patients who received a cementless CBC straight stem (Mathys AG, Bettlach, Switzerland) between 2005 and 2019. We analyzed the migration pattern using the EBRA-FCA software and reviewed their medical histories. In addition, periprosthetic radiolucency was rated according to the Gruen zones and femoral configuration according to Dorr. Results: A total of 333 stems in 332 patients (female 191; male 141) met our inclusion criteria. The mean age at surgery was 63 (range 21–87) years. Migration analysis by EBRA-FCA showed a mean subsidence of 1.6 mm at final follow-up at 96 months with a maximum noted mean subsidence of 2.0 mm at 72 and 84 months. Dorr Type A showed a tendency of less subsidence than did Dorr Type B and was statistically significant at 6 (*p* = 0.0396) and 72 months (*p* = 0.0127). The body mass index (BMI) and increased subsidence were not found to correlate (*p* > 0.05). For this cohort, the overall femoral revision-free rate was 95.2% and the revision-free rate for aseptic loosening was 99.1%. Conclusions: The results showed migration behavior in cementless stems with initial increased migration and subsequent secondary stabilization, suggesting an excellent long-term outcome. Stem migration of this tapered proximally anchored stem might be lower in Dorr Type A than in Dorr Type B femurs without being statistically significant at all time points.

## 1. Introduction

Total hip arthroplasty (THA) is an economical procedure in cases of symptomatic hip osteoarthritis, which brings improvements in quality of life related to health [1,2]. During the last two decades, the number of primary THAs has risen, and projections suggest continued growth in the future [3,4]. Aside from this, the number of revisions has also increased, with aseptic loosening seen to be the most-common cause of implant failure [5,6,7]. Aseptic loosening can result from inadequate initial fixation, mechanical loss of fixation over time, or biological loss of fixation due to particle-induced osteolysis around the implant [8]. Previous studies have reported that distal migration of the stem, also known as subsidence, is a good predictive factor for early aseptic loosening [9,10,11]. In this context, distal migration of the stem of more than 1.5 mm as demonstrated with Einzel Bild Röntgen Analyse-Femoral Component Analysis (EBRA-FCA) within the first two years was indicated by Krismer et al. as a risk factor for early implant failure [12]. However, considering that Krismer et al. included cementless and cemented implants, comparability was limited [12]. In addition, Streit et al. classified a threshold of 2.7 mm axial migration to be critical for the cementless Spotorno (CLS) stem (Zimmer Inc., Warsaw, IN, USA) within the first two years after surgery [11].

EBRA-FCA is a computer-based method for calculating the distal migration of femoral stems using standard anterior–posterior (ap) pelvic radiographs and does not call for additional means at exposure (e.g., ball markers). In comparison to roentgen stereophotogrammetric analysis (RSA), EBRA-FCA has a proven measurement accuracy for detecting axial migration of more than 1 mm, while showing very good interobserver reliability [12,13,14].

The extent of stem subsidence may furthermore be contributed by patient demographics and surgical factors, including BMI, as well as the fit and fill of the stem in relation to the particular femoral configuration [15]. However, available studies that yielded different results depending on the investigated implant have been presented in the past [15,16,17,18,19,20,21,22,23,24,25,26].

The examined stem is the CBC by Mathys (Mathys AG, Bettlach, Switzerland), which has been in clinical use for cementless, press-fit application since 1997 [27]. It has several ribs at the proximal third of the stem following the aim of transferring the load proximally into the bone [27].

The purpose of this study was to investigate the clinical results, as well as the migration behavior of the CBC stem by means of EBRA-FCA with a mid-term follow-up of up to 96 months. Furthermore, we evaluated the possible influence of femoral configuration, body mass index (BMI), and radiolucent lines on stem subsidence.

## 2. Materials and Methods

The study was approved by the local ethics committee (Medical University of Innsbruck, Austria, Europe). This retrospective study looked at all consecutive patients who received a CBC stem at the Department of Orthopaedics and Traumatology of the Medical University of Innsbruck between 2005 and 2019, during which time a total of 1373 CBC stems were implanted.

The CBC stem is a proximally anchored, cementless prosthesis based on Prof. Spotorno’s design and anchorage principles from 1982 [27]. The corundum-blasted surface and prismatic rib geometry aim to promote osseointegration and allow stable stem anchorage. The prosthesis is offered in a total of 13 sizes and three different CCD angles (125°, 135°, 145°). The smaller sizes are available in 1 mm increments and the larger sizes in 1.25 mm or 2.5 mm increments. For the best-possible reconstruction of the offset, a standard and lateralized version is available [27].

The medical histories were examined for sociodemographic data, surgical approach, BMI, cut-to-suture time, material breakage, and preoperative diagnosis for THA indication. Furthermore, the femoral configuration was classified based on the preoperative X-ray according to Dorr (Type A “champagne flute”, Type B “normal”, Type C “stovepipe”) [28]. Moreover, during clinical examination, surgeons at our department determined the range of motion preoperatively and up to one year after surgery using a goniometer. Furthermore, the latest X-ray was examined for radiolucent regions according to the Gruen zones [29].

**Figure 1:**
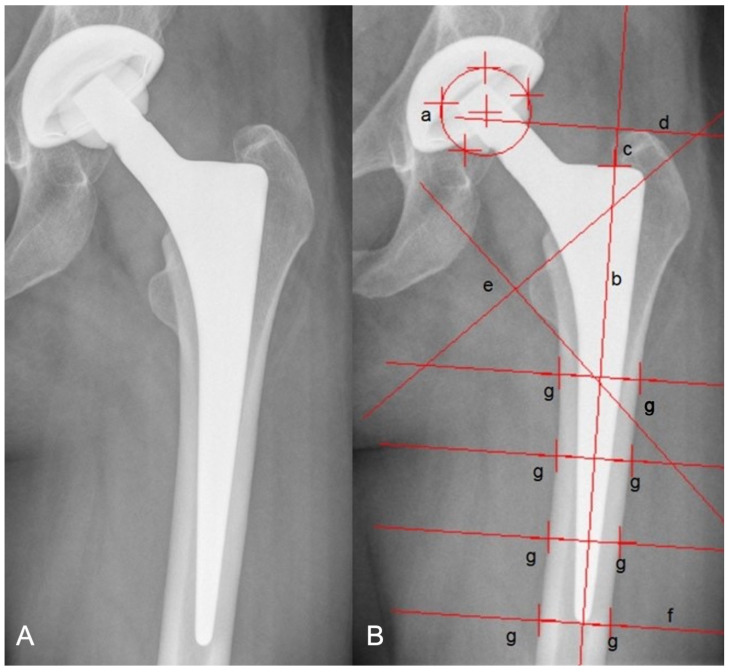
Anterior to posterior X-ray shows a CBC stem (**A**) and one with EBRA-FCA references (**B**): (a) head points, (b) stem axis, (c) stem shoulder, (d) major trochanter line, (e) minor trochanter lines, (f) tip-of-stem line, (g) points at femoral bone contour.

Axial stem migration, prosthetic stability, and stem tilt were evaluated retrospectively with EBRA-FCA and plain X-rays [12,13]. Up to 19 reference points were identified on the femoral head (*n* = 3–7), stem (*n* = 2), femoral cortex (*n* = 8), and 1 at the major and minor trochanter each [13]. The EBRA-FCA software rejects unsuitable X-ray images by means of a comparability algorithm that recognizes significant positioning artifacts by comparing specific bone and prosthetic landmarks [13]. Figure 1 illustrates EBRA-FCA references in an X-ray of a CBC stem.

At the Department of Orthopaedics and Traumatology of the Medical University of Innsbruck, we routinely follow up with radiographs at discharge, 6 weeks after surgery, 12 months postoperative, and then at 1- to 2-year intervals. We take further radiographs if the patient voices complaints with the THA. All X-rays were taken at our Department of Radiology using the same technique (ap radiographs; patient standing in upright position and full weight bearing). Inclusion criteria for our investigation were a minimum of three radiographs per patient and a minimum of five years follow-up. Migration analysis was performed with EBRA-FCA by one independent investigator, who did not play a role in the surgeries or postoperative patient care. Migration was observed in 297 (89.2%) of the 333 included prostheses.

### Statistics

Descriptive statistics (mean, median, range, and standard deviation) were recorded in Excel (Microsoft Excel 2016, Redmond, WA, USA). All calculations for comparative statistics were performed with GraphPad Prism (Version 8.0, GraphPad Software, Inc., La Jolla, CA, USA). The Kolmogorov–Smirnov test was used to examine the normal dataset distribution. As in the majority of the cases the data were not normally distributed, the Kruskal–Wallis test with Dunn’s correction for multiple comparison was used to compare the EBRA-FCA measurements at different time steps. For comparison of the EBRA measurements for patients with and without Gruen zones and divided by their BMI (normal BMI ≤ 25 kg/m^2^, overweight BMI 25.1–29.9 kg/m^2^, and obese BMI ≥ 30 kg/m^2^), the Mann–Whitney U test was used. The range of preoperative and postoperative motion was analyzed using the Wilcoxon matched-pairs signed-rank test. A *p*-value of 0.05 was considered statistically significant.

## 3. Results

A total of 332 patients who underwent THA surgery, met the inclusion criteria, and gave informed consent were included in the study (female: 191; male: 141; ratio 57:43). The mean patient age was 63 (range 21–87) years, and the mean follow-up was eight (range 5–15) years. The mean BMI was 27 (range 16–56) kg/m^2^. Of the patients, 81.1% were scheduled for THA surgery due to primary osteoarthritis (OA), 6.9% due to avascular necrosis of the femoral head, 5.7% due to a dysplastic hip OA of the hip, 1.5% due to an OA with protrusio acetabuli, 1.5% due to a post-traumatic OA, and 3.3% because of other reasons for secondary OA. In 1% of the cases, THA was performed on both sides, in 52% on the right side, and in 47% on the left side. All patients were operated on in supine position except for two cases, the majority (51.7%) with a lateral approach, followed by a direct anterior approach (47.4%) and the dorsal, anterolateral, and extended direct anterior approach (0.3% each). The mean cut-to-suture time was 67 (range 32–253) min. Further details on the patient demographics and surgical procedure are given in Table 1 and details on the implanted stems and cups in Table 2.

EBRA-FCA at eight years follow-up was performed for 297 of the 333 stems with an EBRA-FCA-given comparability limit of 3.0 mm (95% confidence interval). On average, 6.0 (range 3–16) X-rays were analyzed for each implant. A total of 36 patients were excluded from EBRA-FCA migration analysis. A full set of X-ray images at every time step (e.g., 6 months, 12 months, etc.) was not available for the majority of patients, and total subsidence could not be computed for all cases. Due to the drop out of patients during follow-up, a different number of cases in the corresponding migration behavior analysis over time is given.

**Figure 2:**
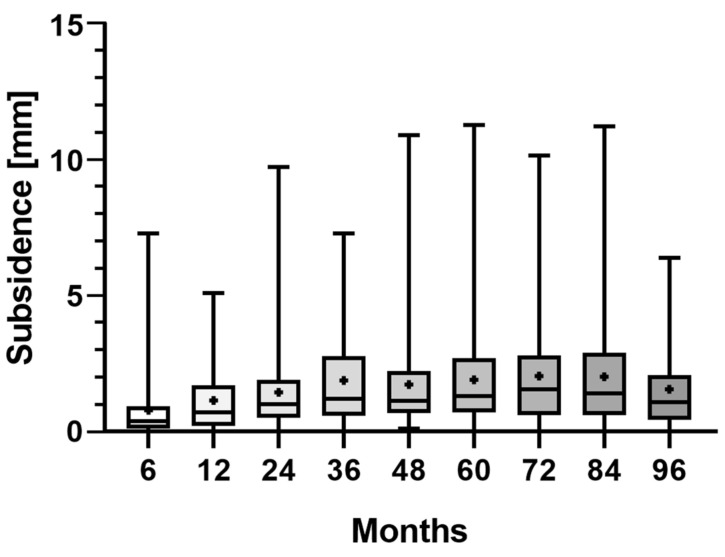
Boxplots of the measured subsidence at clinical follow-up at 96 months. The mean is shown as a plus sign and bars represent the minimum and maximum.

The EBRA-FCA demonstrated a mean migration of 0.8 mm (range 0.0–7.3) at 6 months, 1.1 mm (range 0.0–5.1) at 12 months, 1.4 mm (range 0.0–9.8) at 24 months, 1.9 mm (range 0.0–7.3) at 36 months, 1.7 mm (range 0.1–11.0) at 48 months, 1.9 mm (range 0.0–11.3) at 60 months, 2.0 mm (range 0.0–10.2) at 72 months, 2.0 mm (range 0.0–11.3) at 84 months, and 1.6 mm (range 0.0–6.4) at 96 months of follow-up. A statistically significant difference was observed between subsidence occurring within 6 months and at all other time steps (*p* < 0.0006) except for the period 6–12 months (*p* = 0.1346). However, a statistically significant higher subsidence was found after six years as compared to after one year of follow-up (*p* = 0.0398). Mean monthly axial implant migration was 0.13 mm/month within the first 6 months, 0.06 mm/month between 6 and 12 months, 0.03 mm/month between 12 and 24 months, and 0.04 mm/month between 24 and 36 months after surgery and remained under 0.04 mm/month on average for the rest of the follow-up period of 8 years. Consequently, the main axial subsidence was seen to occur particularly in the first six months following surgery (Figure 2).

**Figure 3:**
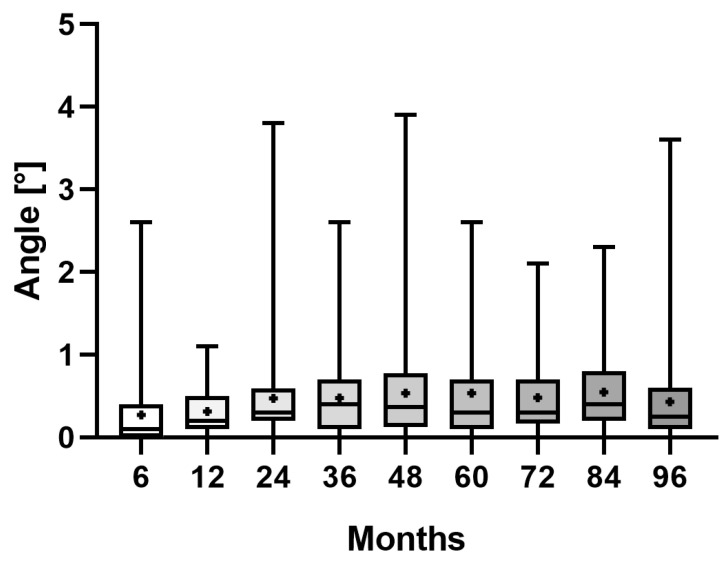
Boxplots of the measured angle between stem and femur axis at clinical follow-up at 96 months. The mean is shown as a plus sign and bars represent the minimum and maximum.

Additionally, the mean angle between the stem and femoral axis measured 0.3° (range 0.0–2.6°) at 6 months, 0.3° (range 0.0–1.1°) at 12 months, 0.5° (range 0.0–3.8°) at 24 months, 0.5° (range 0.0–2.6°) at 36 months, 0.5° (range 0.0–3.9°) at 48 months, 0.5° (range 0.0–2.6°) at 60 months, 0.5° (range 0.0–2.1°) at 72 months, 0.5° (range 0.0–2.3°) at 84 months, and 0.4° (range 0.0–3.6°) at 96 months (Figure 3). A statistically significant difference was established between the angle deviation occurring within 6 months and all other time steps (*p* < 0.0018) except for the period 6–12 months (*p* > 0.9999).

**Figure 4:**
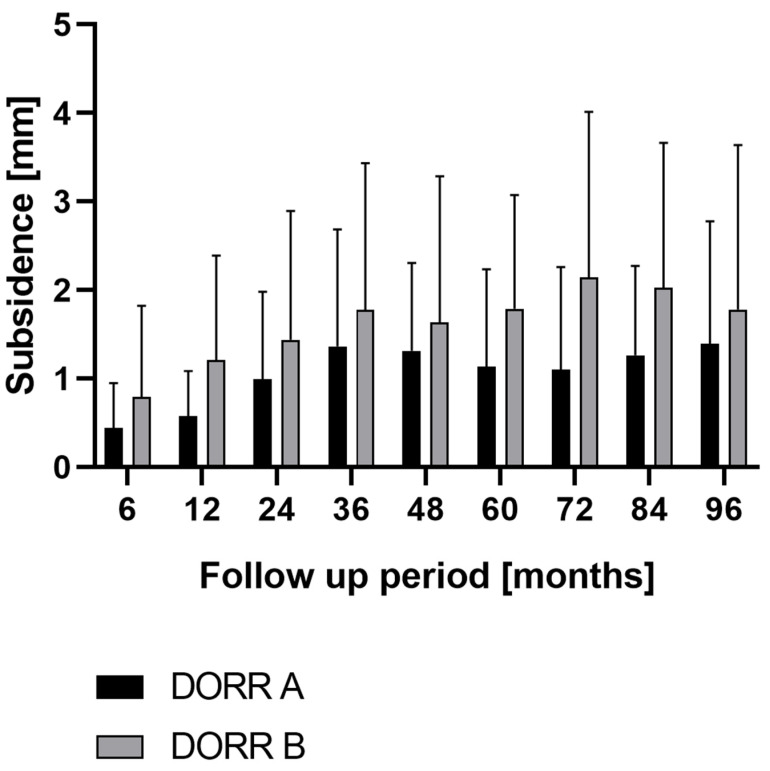
Mean and standard deviation (bars) of total stem subsidence in Dorr Type-A and Dorr Type-B femurs for clinical follow-up at 96 months.

Patients were split into two groups according to the Dorr classification (only one patient was classified as Dorr Type C and was excluded from the analysis). A statistically significant lower mean subsidence of 0.44 mm was observed for patients classified with a Dorr Type A within six months of follow-up as compared to patients classified with a Dorr Type B (mean subsidence 0.79, *p* = 0.0396). After six years, a statistically significant lower mean subsidence of 1.1 was observed for patients classified with a Dorr Type A as compared to patients classified with a Dorr Type B (mean subsidence 2.14, *p* = 0.0127) (Figure 4).

**Figure 5:**
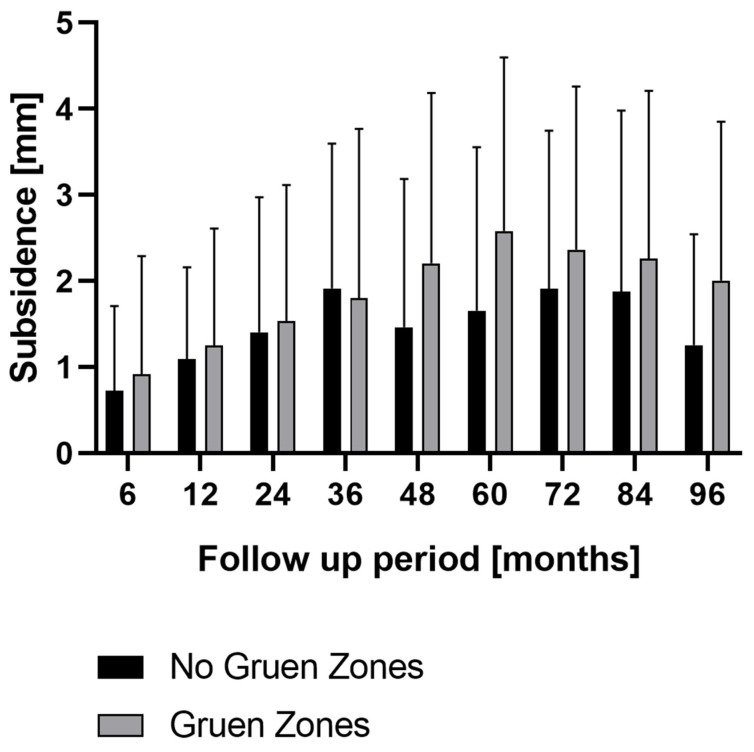
Mean and standard deviation (bars) of total stem subsidence in cases where radiolucent lines were found in Gruen zones or not at clinical follow-up at 96 months.

The final radiograph for each patient in our study group was examined for radiolucent regions according to the Gruen zones, and in 96 (28.8%) patients, a radiolucent margin in at least one of the Gruen zones was found. This was most often the case in Gruen Zones 1 (50.6%) and 7 (20.9%). We divided our patient cohort into two groups according to the Gruen zones in order to measure the effect on subsidence: patients with or without radiolucent lines in the Gruen zones. There was no statistically significant difference in subsidence between the two sub-cohorts except for the follow-up period of five years, where a statistically significant higher subsidence was observed for the group with Gruen zones than for the group without Gruen zones (mean 2.6 with Gruen zones versus 1.7 without Gruen zones, *p* = 0.0080) (Figure 5).

Additionally, the patients were divided into groups according to their BMI: normal (BMI ≤ 25 kg/m^2^), overweight (BMI 25.1–29.9 kg/m^2^), and obese (BMI ≥ 30 kg/m^2^). No statistically significant difference was found between the three groups when considering the subsidence for the follow-up period (*p* > 0.05).

Comparison of the range of motion preoperatively and postoperatively showed a statistically significant mean improvement in flexion of 7° (*p* < 0.0001), a mean improvement in internal rotation of 11° (*p* < 0.0001), a mean improvement of 9° in external rotation (*p* < 0.0001), as well as a mean improvement of 8° in adduction (*p* < 0.0001) and 7° in abduction (*p* < 0.0001).

A total of 16 stems (4.8%) from the overall cohort required stem revision, of which 7 (2.1%) were due to periprosthetic infection, 6 (1.8%) due to periprosthetic fracture, and only 3 (0.9%) due to aseptic loosening. In one case of aseptic loosening, the stem showed progressive subsidence from the onset with a 24-month subsidence of 6.5 mm. The largest subsidence of 11.3 mm was observed in a patient who suffered a periprosthetic fracture one month after primary implantation, which was treated with cerclages without stem replacement. No further subsidence was observed after the 48-month measurement period, and no complaints were documented on the part of the patient. No case of material breakage was detected. This gives an overall stem revision-free rate of 95.2% and a revision-free rate for aseptic loosening of 99.1% for this cohort.

## 4. Discussion

In this study, we examined the migration pattern of the proximally anchored CBC (Mathys AG, Bettlach, Switzerland) straight stems. The most-important finding was the fact that mean migration in the study group was 1.4 mm at 24 months and 1.6 mm at last follow-up at 96 months.

A high degree of early stability is known to be a key factor for rapid osseointegration in primary THA [30]. Based on this, increased subsidence of the femoral component during the first two years after implantation is considered to be an important risk factor correlating with subsequent aseptic loosening [11,12]. With a specificity of 100% and a sensitivity of 78% for detection of migration of more than 1 mm as compared with RSA, EBRA-FCA can be said to be suitable for demonstrating and measuring the subsidence of femoral components in THA [13]. While RSA is classified as the gold standard for migration measurements, it requires the implantation of tantalum marker balls, making it applicable only in prospective study designs [31]. The advantage of EBRA-FCA is that it is a non-invasive method that can be applied in our retrospective study design.

The current literature offers various thresholds for the prediction of aseptic loosening [9,11,12]. Already in 1994, Freemann et al. reported a cut-off value for subsidence of 1.2 mm per year within the first two years after surgery for predicting aseptic failure with a sensitivity of 78% [9]. Parallel with this, Krismer et al. reported a migration of more than 1.5 mm within two years postoperatively detected with EBRA-FCA as being predictive for subsequent implant failure with a sensitivity of 69% [12]. However, it should be noted that Freeman et al., as well as Krismer et al. evaluated a heterogeneous cohort of cemented and cementless stems [9,12]. Furthermore, Streit et al. presented a relatively high threshold of 2.7 mm at two years postoperative measured with EBRA-FCA as having the best diagnostic performance (sensitivity 56%, specificity 99%) for the prediction of the aseptic loosening of the cementless CLS stem (Zimmer Inc, Warsaw, IN, USA) [11].

Different types of cementless stems have been investigated for migration using various measurement techniques. In a prospective randomized trial, Reiner et al. calculated a mean migration of 1.08 mm (SD 0.93 mm) by RSA for the SL-PLUS MIA stem (Smith & Nephew Orthopaedics AG, Baar, Switzerland) after two years with subsidence mainly occurring during the first six weeks after surgery [32]. While an EBRA-FCA by Stihsen et al. showed a mean subsidence of 1.38 mm for 105 cementless Vision 2000 stems (DePuy, Warsaw, IN, USA) two years postoperative, the study by Dammerer et al. yielded a mean migration of 2.2 mm for the collarless Corail stem (DePuy Orthopaedics Inc., Warsaw, IN, USA) after two years as measured with the same technique [16,33]. Ström et al. identified a mean subsidence of 1.2 mm for the cementless CLS stem (Centerpulse, Bern, Switzerland) after two years by RSA [34]. The current EBRA-FCA of the CBC stem showed a mean migration of 1.4 mm two years after surgery and 1.6 mm after eight years. Our results are well in line with the aforementioned studies and below the thresholds referenced by Krismer et al. and Streit et al. [11,12]. The main subsidence occurred during the initial six months with a subsequent reduction in the migration rate.

A number of studies have already investigated the potential of BMI and weight as a factor affecting stem loosening and revealed different findings for different stem types [15,16,17,18,19,20,21,22,23]. According to Bergmann et al., the levels of contact forces, as well as torsional moments are determined by the BMI and influence the femoral stem during daily activities [35]. Moreover, a higher BMI was reported to significantly increase the risk of stem loosening, namely by 2.6% per additional unit of BMI, whereby neither weight nor height was seen to be a significant predictor of stem loosening in a case–control study of 5035 patients [36]. However, body weight over 75 kg was seen to have a significant impact on subsidence of the Vision 2000 stem (DePuy, Warsaw, IN, USA) as observed by Stihsen et al., whereas a BMI > 30 kg/m^2^ had no influence [16]. In contrast, the findings of Akram et al. showed increased BMI to be independently associated with an increased risk for subsidence of a trabecular metal taper femoral prosthesis, for which reason the authors recommended caution in utilizing this stem in obese patients [22]. In the study by Grant et al., a significant increase in subsidence was noted for fit-and-fill stems with increasing BMI (*p* = 0.001), while this relationship was not found for tapered wedge design stems (*p* = 0.013) [21]. The study group argued that the resistance to subsidence, regardless of BMI, is likely due to the inherent axial stability of a tapered wedge design, which may represent the best stem design for obese patients [21]. In our study, no significant differences in subsidence was seen between normal, overweight, and obese patients, and thus, this stem may be a suitable therapeutic option in overweight and obese patients.

In addition to patient demographics, initial press fit is an essential determinant for primary stability [37]. Therefore, different femoral configurations such as “champagne flute”, “normal”, or “stovepipe” have been described and should be considered when choosing the stem type [28,38]. A relationship between femoral configuration and axial migration has been described in the past [24,25]. In the current study, mean subsidence at six months (*p* = 0.0396), as well as six years (*p* = 0.0127) was significantly lower in Dorr Type-A than in Dorr Type-B femurs, with a trend toward lower subsidence in Dorr Type A throughout the whole study period without statistical significance at any time point. Park et al. examined stem survival of the cementless Bencox stem (Corentec, Cheon-An, South Korea) over a minimum follow-up of five years [26]. Contrarily, stem survival turned out to be significantly lower in Dorr Type-A than in Dorr Type-B femurs (*p* = 0.041) [26]. However, the predominant reason for stem revision in Type-A femurs was periprosthetic fracture (67%), followed by aseptic loosening (22%) and deep infection (11%) [26].

Another radiological criterion often used to determine stem stability is the appearance of radiolucent lines [39]. Pospischill et al., therefore, investigated the Alloclassic SL Stem (Zimmer/Centerpulse, Winterthur, Switzerland), which has a diaphyseal press-fit fixation [40]. Radiolucent lines appeared in Gruen Zone 1 in 50.5% of the cases and in Gruen Zone 7 in 25.2% of the cases, with no progression after the first two years [40]. A reason for the radiolucent lines was suspected to be proximal micromotion while the stem is distally fixed, but might also result from an intraoperative change in the primary rasping direction [41]. In the study by Zang et al., radiolucent lines appeared in 39.7% of the investigated CLS stems (Zimmer Inc., Warsaw, IN, USA) [42]. In anteroposterior X-rays, radiolucent lines were limited to Gruen Zones 1 and 7 [42]. The authors suggested that radiolucent lines observed mainly at the proximal femur resulted from wear particles, which did not compromise stem stability [42]. Already in 2002, Grappiolo et al. pointed out that non-progressive radiolucent lines in one to three Gruen zones around the stem did not affect the stability of the prosthesis at long-term follow-up [43]. In the present study, radiolucent lines at last follow-up were observed in 28.8% of CBC stems and were mostly detected in Gruen Zones 1 (50.6%) and 7 (20.9%), which is well in line with the previously mentioned literature. No statistically significant difference in subsidence was found, except for the follow-up period of five years, where the reduction was greater in the group with detected radiolucency.

Postoperative periprosthetic femoral fracture is another reason that may be associated with stem loosening [44,45]. Following THA, the risk of periprosthetic fracture is estimated to be 0.4–3.5% [45,46]. In the present study, the largest stem subsidence was observed as a result of a periprosthetic fracture occurring within the first month after primary THA, which was stabilized with cerclages without stem revision. While the subsidence was 9.8 mm after 24 months, it subsequently remained stable at about 11 mm after 48 months. As the patient had no complaints, no further revision was performed. This shows that, although there are several classifications that can be used as a guide for the general treatment of periprosthetic fractures, it is important for the surgeon to understand that the treatment needs to be individualized, influenced by different factors [47].

The present study had some limitations that need to be mentioned. First, this study had a retrospective design and no control group. As a result of its retrospective study design, some of the patients had to be excluded from the cohort, thus possibly making the study more susceptible to selection bias. Second, the different number of X-ray images of each hip made during the follow-up, combined with the smoothing function of EBRA-FCA, may have influenced migration results. Third, as stem migration is multifactorially influenced, not all factors could be ruled out, and some patient characteristics (e.g., osteoporosis, smoking) could not be assessed. Fourth, no specific hip score for investigating the clinical outcome was available. Fifth, a predictive model was not established due to a too-short follow-up time with a limited number of detected revisions due to aseptic loosening.

## 5. Conclusions

In summary, EBRA-FCA for the cementless CBC straight stem showed an acceptable mean subsidence in accordance with known thresholds at final follow-up. Implant position stabilized after initial subsidence. Therefore, the revision-free rate for aseptic loosening was excellent at 99.1%. BMI had no significant effect on stem subsidence.

## Figures and Tables

**Table 1:** Demographics of the study group. Range is given in brackets.

Number of Patients	Female	191
	Male	141
	Total	332
Surgical site	Left	176
	Right	157
Mean age (years)		63 [21–87]
Mean radiological follow-up (years)		8 [5–15]
BMI (kg/m^2^)		27 [16–56]
Surgical approach	Lateral approach	172
	Direct anterior approach	158
	Extended direct anterior approach	1
	Posterior approach	1
	Anterolateral approach	1
Surgical position	Supine	331
	Lateral	2
Preoperative diagnosis	Primary osteoarthritis	270
	Avascular necrosis of femoral head	23
	Dysplastic hip	19
	Protrusio acetabuli	5
	Post-traumatic osteoarthritis	5
	Other secondary osteoarthritis	11

**Table 2:** Details on Dorr classification and implanted components. Percentage given in brackets.

Dorr Classification	A	159 [47.7]
	B	173 [52.0]
	C	1 [0.3]
CBC stem design	Standard	216 [64.9]
	Lateral	117 [35.1]
Head size (mm)—ceramic	28	70 [21.0]
	32	262 [78.7]
	36	1 [0.3]
Cup	Duraloc	210 [63.1]
	Trident	93 [27.9]
	Pinnacle	15 [4.5]
	Other cups	15 [4.5]

## Data Availability

Data available on request due to restrictions, e.g., privacy or ethical. The data presented in this study are available on request from the corresponding author.

## References

[1] Ethgen O., Bruyère O., Richy F., Dardennes C., Reginster J.-Y. (2004). Health-Related Quality of Life in Total Hip and Total Knee Arthroplasty. A Qualitative and Systematic Review of the Literature. J. Bone Jt. Surg. Am..

[2] Pivec R., Johnson A.J., Mears S.C., Mont M.A. (2012). Hip Arthroplasty. Lancet.

[3] Pabinger C., Lothaller H., Portner N., Geissler A. (2018). Projections of Hip Arthroplasty in OECD Countries up to 2050. Hip. Int..

[4] Singh J.A., Yu S., Chen L., Cleveland J.D. (2019). Rates of Total Joint Replacement in the United States: Future Projections to 2020-2040 Using the National Inpatient Sample. J. Rheumatol..

[5] Haynes J.A., Stambough J.B., Sassoon A.A., Johnson S.R., Clohisy J.C., Nunley R.M. (2016). Contemporary Surgical Indications and Referral Trends in Revision Total Hip Arthroplasty: A 10-Year Review. J. Arthroplast..

[6] Feng X., Gu J., Zhou Y. (2022). Primary Total Hip Arthroplasty Failure: Aseptic Loosening Remains the Most Common Cause of Revision. Am. J. Transl. Res..

[7] Kurtz S., Ong K., Lau E., Mowat F., Halpern M. (2007). Projections of Primary and Revision Hip and Knee Arthroplasty in the United States from 2005 to 2030. J. Bone Jt. Surg. Am..

[8] Anil U., Singh V., Schwarzkopf R. (2022). Diagnosis and Detection of Subtle Aseptic Loosening in Total Hip Arthroplasty. J. Arthroplast..

[9] Freeman M.A., Plante-Bordeneuve P. (1994). Early Migration and Late Aseptic Failure of Proximal Femoral Prostheses. J. Bone Jt. Surg. Br..

[10] Kroell A., Beaulé P., Krismer M., Behensky H., Stoeckl B., Biedermann R. (2009). Aseptic Stem Loosening in Primary THA: Migration Analysis of Cemented and Cementless Fixation. Int. Orthop..

[11] Streit M.R., Haeussler D., Bruckner T., Proctor T., Innmann M.M., Merle C., Gotterbarm T., Weiss S. (2016). Early Migration Predicts Aseptic Loosening of Cementless Femoral Stems: A Long-Term Study. Clin. Orthop. Relat. Res..

[12] Krismer M., Biedermann R., Stöckl B., Fischer M., Bauer R., Haid C. (1999). The Prediction of Failure of the Stem in THR by Measurement of Early Migration Using EBRA-FCA. Einzel-Bild-Roentgen-Analyse-Femoral Component Analysis. J. Bone Jt. Surg. Br..

[13] Biedermann R., Krismer M., Stöckl B., Mayrhofer P., Ornstein E., Franzén H. (1999). Accuracy of EBRA-FCA in the Measurement of Migration of Femoral Components of Total Hip Replacement. Einzel-Bild-Röntgen-Analyse-Femoral Component Analysis. J. Bone Jt. Surg. Br..

[14] Biedermann R., Stöckl B., Krismer M., Mayrhofer P., Ornstein E., Franzén H. (2001). Evaluation of Accuracy and Precision of Bone Markers for the Measurement of Migration of Hip Prostheses. A Comparison of Conventional Measurements. J. Bone Jt. Surg. Br..

[15] Nazari-Farsani S., Vuopio M., Löyttyniemi E., Aro H.T. (2021). Contributing Factors to the Initial Femoral Stem Migration in Cementless Total Hip Arthroplasty of Postmenopausal Women. J. Biomech..

[16] Stihsen C., Radl R., Keshmiri A., Rehak P., Windhager R. (2012). Subsidence of a Cementless Femoral Component Influenced by Body Weight and Body Mass Index. Int. Orthop..

[17] Ibrahim T., Hobson S., Beiri A., Esler C.N. (2005). No Influence of Body Mass Index on Early Outcome Following Total Hip Arthroplasty. Int. Orthop. SICO.

[18] Liu W., Wahafu T., Cheng M., Cheng T., Zhang Y., Zhang X. (2015). The Influence of Obesity on Primary Total Hip Arthroplasty Outcomes: A Meta-Analysis of Prospective Cohort Studies. Orthop. Traumatol. Surg. Res..

[19] Melloh M., Eggli S., Busato A., Roder C. (2011). Predictors of Early Stem Loosening after Total Hip Arthroplasty: A Case-Control Study. J. Orthop. Surg..

[20] Freitag T., Kappe T., Fuchs M., Jung S., Reichel H., Bieger R. (2014). Migration Pattern of a Femoral Short-Stem Prosthesis: A 2-Year EBRA-FCA-Study. Arch. Orthop. Trauma. Surg..

[21] Grant T.W., Lovro L.R., Licini D.J., Warth L.C., Ziemba-Davis M., Meneghini R.M. (2017). Cementless Tapered Wedge Femoral Stems Decrease Subsidence in Obese Patients Compared to Traditional Fit-and-Fill Stems. J. Arthroplast..

[22] Akram F., Kunze K.N., Kerzner B., Gonzalez A., Palacios A., Levine B.R. (2021). Mid-Term Survivorship, Performance, and Predictors of Outcome in Primary Total Hip Arthroplasty With a Porous Tantalum Femoral Prosthesis. J. Arthroplast..

[23] Al-Najjim M., Khattak U., Sim J., Chambers I. (2016). Differences in Subsidence Rate between Alternative Designs of a Commonly Used Uncemented Femoral Stem. J. Orthop..

[24] White C.A., Carsen S., Rasuli K., Feibel R.J., Kim P.R., Beaulé P.E. (2012). High Incidence of Migration with Poor Initial Fixation of the Accolade Stem. Clin. Orthop. Relat. Res..

[25] Jahnke A., Engl S., Seeger J.B., Basad E., Rickert M., Ishaque B.A. (2015). Influences of Fit and Fill Following Hip Arthroplasty Using a Cementless Short-Stem Prosthesis. Arch Orthop. Trauma. Surg..

[26] Park C.-W., Eun H.-J., Oh S.-H., Kim H.-J., Lim S.-J., Park Y.-S. (2019). Femoral Stem Survivorship in Dorr Type A Femurs after Total Hip Arthroplasty Using a Cementless Tapered Wedge Stem: A Matched Comparative Study With Type B Femurs. J. Arthroplast..

[27] Mathys CBC Surgical Technique/Product Information 2020. https://www.mathysmedical.com/Storages/User/Dokumente/Operationstechnik/Huefte/OP-Technik_Produktinfo_CBC_short-cone_DE_V05.pdf.

[28] Dorr L.D., Faugere M.C., Mackel A.M., Gruen T.A., Bognar B., Malluche H.H. (1993). Structural and Cellular Assessment of Bone Quality of Proximal Femur. Bone.

[29] Gruen T.A., McNeice G.M., Amstutz H.C. (1979). “Modes of Failure” of Cemented Stem-Type Femoral Components: A Radiographic Analysis of Loosening. Clin. Orthop. Relat. Res..

[30] Karuppal R. (2016). Biological Fixation of Total Hip Arthroplasty: Facts and Factors. J. Orthop..

[31] Selvik G. (1990). Roentgen Stereophotogrammetric Analysis. Acta Radiol..

[32] Reiner T., Sonntag R., Kretzer J.P., Clarius M., Jakubowitz E., Weiss S., Ewerbeck V., Merle C., Moradi B., Kinkel S. (2020). The Migration Pattern of a Cementless Hydroxyapatite-Coated Titanium Stem under Immediate Full Weight-Bearing-A Randomized Controlled Trial Using Model-Based RSA. J. Clin. Med..

[33] Dammerer D., Blum P., Putzer D., Krappinger D., Pabinger C., Liebensteiner M.C., Thaler M. (2022). Migration Characteristics of the Corail Hydroxyapatite-Coated Femoral Stem—A Retrospective Clinical Evaluation and Migration Measurement with EBRA. Arch. Orthop. Trauma. Surg..

[34] Ström H., Nilsson O., Milbrink J., Mallmin H., Larsson S. (2007). The Effect of Early Weight Bearing on Migration Pattern of the Uncemented CLS Stem in Total Hip Arthroplasty. J. Arthroplast..

[35] Bergmann G., Deuretzbacher G., Heller M., Graichen F., Rohlmann A., Strauss J., Duda G.N. (2001). Hip Contact Forces and Gait Patterns from Routine Activities. J. Biomech..

[36] Münger P., Röder C., Ackermann-Liebrich U., Busato A. (2006). Patient-Related Risk Factors Leading to Aseptic Stem Loosening in Total Hip Arthroplasty: A Case-Control Study of 5035 Patients. Acta Orthop..

[37] Viceconti M., Brusi G., Pancanti A., Cristofolini L. (2006). Primary Stability of an Anatomical Cementless Hip Stem: A Statistical Analysis. J. Biomech..

[38] Noble P.C., Alexander J.W., Lindahl L.J., Yew D.T., Granberry W.M., Tullos H.S. (1988). The Anatomic Basis of Femoral Component Design. Clin. Orthop. Relat. Res..

[39] Meding J.B., Ritter M.A., Keating E.M., Faris P.M. (1994). Clinical and Radiographic Evaluation of Long-Stem Femoral Components Following Revision Total Hip Arthroplasty. J. Arthroplast..

[40] Pospischill M., Knahr K. (2005). Cementless Total Hip Arthroplasty Using a Threaded Cup and a Rectangular Tapered Stem. Follow-up for Ten to 17 Years. J. Bone Jt. Surg. Br..

[41] Rivière C., Grappiolo G., Engh C.A., Vidalain J.-P., Chen A.-F., Boehler N., Matta J., Vendittoli P.-A. (2018). Long-Term Bone Remodelling around “legendary” Cementless Femoral Stems. EFORT Open. Rev..

[42] Zang J., Uchiyama K., Moriya M., Li Z., Fukushima K., Yamamoto T., Takahira N., Takaso M., Liu J., Feng W. (2018). Long-Term Clinical and Radiographic Results of the Cementless Spotorno Stem in Japanese Patients: A More than 15-Year Follow-Up. J. Orthop. Surg..

[43] Grappiolo G., Blaha J.D., Gruen T.A., Burastero G., Spotorno L. (2002). Primary Total Hip Arthroplasty Using a Grit-Blasted, Press-Fit Femoral Prosthesis.Long-Term Results with Survivorship Analysis. Hip. Int..

[44] Mondanelli N., Troiano E., Facchini A., Ghezzi R., Di Meglio M., Nuvoli N., Peri G., Aiuto P., Colasanti G.B., Giannotti S. (2022). Treatment Algorithm of Periprosthetic Femoral Fracturens. Geriatr. Orthop. Surg. Rehabil..

[45] Lindahl H., Malchau H., Herberts P., Garellick G. (2005). Periprosthetic Femoral Fractures Classification and Demographics of 1049 Periprosthetic Femoral Fractures from the Swedish National Hip Arthroplasty Register. J. Arthroplast..

[46] Abdel M.P., Watts C.D., Houdek M.T., Lewallen D.G., Berry D.J. (2016). Epidemiology of Periprosthetic Fracture of the Femur in 32,644 Primary Total Hip Arthroplasties: A 40-Year Experience. Bone Jt. J..

[47] Patsiogiannis N., Kanakaris N.K., Giannoudis P.V. (2021). Periprosthetic Hip Fractures: An Update into Their Management and Clinical Outcomes. EFORT Open. Rev..

